# Cerebrospinal fluid markers for synaptic function and Alzheimer type changes in late life depression

**DOI:** 10.1038/s41598-021-99794-9

**Published:** 2021-10-13

**Authors:** Nikias Siafarikas, Bjørn-Eivind Kirsebom, Deepak P. Srivastava, Cecilia M. Eriksson, Eirik Auning, Erik Hessen, Geir Selbaek, Kaj Blennow, Dag Aarsland, Tormod Fladby

**Affiliations:** 1 Department of Geriatric Psychiatry, Akershus University Hospital, Sykehusveien 25, 1478 Lørenskog, Norway; 2 Faculty of Medicine, University of Oslo, Oslo, Norway; 3 Department of Neurology, University Hospital of North Norway, Tromsø, Norway; 4 Department of Psychology, Faculty of Health Sciences, UiT The Arctic University of Norway, Tromsø, Norway; 5 Department of Basic and Clinical Neuroscience, Institute of Psychiatry, Psychology and Neuroscience, King’s College London, London, SE5 9NU UK; 6 Department of Neurology, Akershus University Hospital, Lørenskog, Norway; 7 Department of Psychology, University of Oslo, Oslo, Norway; 8 Norwegian National Advisory Unit On Aging and Health, Vestfold Hospital Trust, Tønsberg, Norway; 9 Department of Geriatric Medicine, Oslo University Hospital, Oslo, Norway; 10 Department of Psychiatry and Neurochemistry, Institute of Neuroscience and Physiology, The Sahlgrenska Academy, University of Gothenburg, Gothenburg, Sweden; 11 Clinical Neurochemistry Laboratory, Sahlgrenska University Hospital, Mölndal, Sweden; 12 Department of Old Age Psychiatry, Institute of Psychiatry, Psychology and Neuroscience, King’s College London, London, SE5 8AF UK; 13 Institute of Clinical Medicine, University of Oslo, Campus Ahus, Oslo, Norway

**Keywords:** Biomarkers, Diagnostic markers, Predictive markers, Prognostic markers, Psychiatric disorders, Depression, Diseases, Neurological disorders, Dementia, Alzheimer's disease

## Abstract

To explore markers for synaptic function and Alzheimer disease (AD) pathology in late life depression (LLD), predementia AD and normal controls (NC). A cross-sectional study to compare cerebrospinal fluid (CSF) levels of neurogranin (Ng), Beta-site amyloid-precursor-protein cleaving enzyme1 (BACE1), Ng/BACE1 ratio and Amyloid-β 42/40 ratio, phosphorylated-tau and total-tau in LLD with (LLD AD) or without (LLD NoAD) AD pathology, predementia AD and normal controls (NC). We included 145 participants (NC = 41; predementia AD = 66 and LLD = 38). LLD comprised LLD AD (n = 16), LLD NoAD (n = 19), LLD with non-AD typical changes (n = 3, excluded). LLD AD (p_ADJ_ < 0.05) and predementia AD (p_ADJ_ < 0.0001) showed significantly higher Ng than NC. BACE1 and Ng/BACE1 ratio were altered similarly. Compared to LLD NoAD, LLD AD showed significantly higher Ng (p_ADJ_ < 0.001), BACE1 (p_ADJ_ < 0.05) and Ng/BACE1 ratio (p_ADJ_ < 0.01). All groups had significantly lower Aβ 42/40 ratio than NC (predementia AD and LLD AD, *p* < 0.0001; LLD NoAD, *p* < 0.05). Both LLD groups performed similarly on tests of memory and executive function, but significantly poorer than NC. Synaptic function in LLD depended on AD pathology. LLD showed an association to Amyloid dysmetabolism. The LLD groups performed poorer cognitively than NC. LLD AD may be conceptualized as “predementia AD with depression”.

## Introduction

Late life depression (LLD) is a burden for patients, relatives and healthcare-systems^1^ with high treatment resistance^2^ and poorly understood mechanisms. Clinically, LLD is often hard to separate from Alzheimer’s disease in early, predementia stages (predementia AD) which is defined as biomarker evidence of AD pathology with cognitive impairment but no dementia^3^. An association of LLD to AD pathology such as amyloid plaques has been hypothesized^4^ but results are inconsistent^5^ and a recent study performing Amyloid-β (Aβ) positron emission tomography (PET)^6^ in patients with LLD failed to provide evidence for an association between Aβ and LLD. Synaptic loss and dysfunction are an early feature of AD and tightly connected to amyloid plaque formation^7,8^: the presynaptic Beta-site amyloid-precursor-protein (AβPP) cleaving enzyme 1 (BACE1) instigates the formation of Amyloid-β (Aβ) species. Innate immune activation is involved in BACE1 activation and synaptic loss is likely linked to the release of the postsynaptic protein neurogranin (Ng)^9–11^. Ng is mainly expressed in dendritic spines in the neocortex and the hippocampus^12^ and is associated to cognitive function and memory potentiation^13,14^. Elevated CSF levels of Ng and BACE1 are regarded specific for AD^13,15^ already in predementia stages. Although predementia AD and LLD show a considerable clinical overlap, it is unclear if LLD shows overlapping AD specific constellations of Ng and BACE1. Constellations of Ng and BACE1 in LLD similar to those in AD could indicate common biological underpinnings of the two conditions and might in part explain cognitive symptoms in LLD. Lower levels of Ng and BACE1 in LLD than in AD have been reported in two studies^16,17^. Only one of those studies^16^, though, included normal controls (NC) and found no significant difference in CSF Ng levels between NC and LLD, while both NC and LLD had lower Ng levels than AD. However, neither study defined normal or pathological AD status in their LLD groups. To better study synaptic function and AD pathology in LLD, an approach is needed where LLD patients are stratified according to presence or absence of AD pathology. This would provide the opportunity to study markers for synaptic function in LLD unbiased from possible AD pathology. To the best of our knowledge, no such study has been performed.

To study the relationship between LLD, synaptic function, AD pathology and cognitive performance we included patients with LLD, predementia AD and NC. We expected to find some LLD patients with normal AD biomarkers similar to NC and others with signs of AD pathology as in predementia AD. Our main hypothesis was that synaptic function measured with Ng, BACE1 and Ng/BACE1 ratio levels in LLD depends onthe status of AD biomarkers in LLD: LLD with normal AD biomarkers (LLD NoAD) would show Ng, BACE1 and Ng/BACE1 ratio levels similar to NC; LLD with signs of AD pathology (LLD AD) would show higher Ng, BACE1 and Ng/BACE1 levels than NC as is typical for AD. Moreover, we wanted to compare cognitive performance between the included groups and expected LLD patients to perform poorer than NC.

## Material and methods

### Cohorts and recruitment

The study was based on the Norwegian multicentre project “Dementia Disease Initiation (DDI)”^18^, including participants from a neurocognitive (“DDI cog”) and a neuropsychiatric (“DDI plus”) sub cohort. The study has been approved by the ethics committee “Regional Committees for Medical and Health Research Ethics (REK)”. All participants gave their informed consent in writing. All methods were performed in accordance with relevant REK guidelines and regulations and in accordance with the Declaration of Helsinki.

The “DDI cog” recruited participants from memory clinics and through media. Inclusion criteria for patients in DDI cog were subjective cognitive decline (SCD) and mild cognitive impairment (MCI). The “DDI plus” recruited patients from the Department for old age psychiatry, Akershus University-Hospital; inclusion criteria in DDI plus were psychiatric diagnoses based on ICD-10. Cognitively healthy NC were included from spouses of patients and from orthopaedic patients who underwent surgery in spinal anaesthesia, with CSF collected before the injection of the anaesthetic. For neuropsychological assessment of NC and patients see below.

Exclusion criteria in both cohorts were dementia or severe physical illness. In the DDI cog cohort psychiatric illness such as a diagnosis of depression, bipolar disorder or schizophrenia, which may impair cognition were exclusion criteria. Participants from both cohorts underwent medical examination, neuropsychological assessment (see below), collection of plasma and CSF samples according to study protocol within 3 months from inclusion^18^.

### Diagnosis of LLD

Diagnoses in DDI plus were made according to ICD-10^19^. In addition to clinical examination by a consultant psychiatrist or psychologist, structured diagnostic procedure included the Mini-International Neuropsychiatric Interview (M.I.N.I.)^20^, the neuropsychiatric inventory questionnaire (NPI-Q)^21^ and the Geriatric depression scale (GDS)^22^. The clinical diagnoses were discussed in multidisciplinary consensus meetings including consultant psychiatrists, psychologists and neuropsychologists. In this study we included patients with a diagnosis of depression according to ICD-10 (F32)^23^. There is no defined age cut-off for LLD, but patients in the department for old-age psychiatry are usually 65 years or older. To describe depression in this patient group as LLD was in line with common practice in most studies into LLD where patients with LLD are 60 to 65 and older^24^. In addition, we reported information on the prevalence of Late-onset depression (LOD) (first occurrence after 60 years of age^25^) and comorbid anxiety (in accordance with ICD-10 F41^23^) in the LLD groups.

### Neuropsychological assessment

Neuropsychological assessment in addition to clinical examination was done to stage participants as NC, SCD or MCI based on demographically adjusted *T*-scores from neuropsychological tests (see below) according to published criteria^26,26^. We applied the “Mini Mental State Examination (MMSE-NR)”^28^ and the clock-drawing-test^29^ for cognitive screening; tests on verbal learning and memory recall (Consortium to Establish a Registry for Alzheimer’s Disease [CERAD] word list test)^30^; psychomotor speed and divided attention (trail-making test A and B [TMT A and B])^31^; verbal fluency (Controlled Oral Word Association Test [COWAT])^32^; visual object and space perception (VOSP)^33^. Demographically adjusted T-scores of CERAD word list (recall)^34^, VOSP^33^, TMT B and COWAT^35^ were used for cognitive staging, defined as impaired if lower than 1.5 standard deviation below the normal population. Details on the neuropsychological battery have been provided elsewhere^36^.

### CSF analysis and AD pathology

Lumbar puncture procedures and CSF handling were performed as described in^18^. The QuickPlex SQ 120 system from MesoScale Discovery (MSD, MD, USA) was used to measure Aβ42 and Aβ40 in a multiplex setup using V-plex Ab Peptide Panel 1 (6E10) kit (K15200E-1). T-tau and P-tau concentrations were measured with Innotest kits (Fujirebio, Ghent, Belgium); Ng and BACE1 with Euroimmun AG kits (Luebeck, Germany)^37^.

AD pathology was assessed according to normal or pathological Amyloid (A), Phosphorylated (P)-tau (T) and total (T)-tau (N) (introduced as A/T/N index in^38^). To define pathological Aβ levels (A) we used the Aβ42/40 ratio, which has been suggested superior to Aβ42 alone to distinguish AD^39^. An optimum cut-off for Aβ42/40 ratio at ≤ 0.077 was determined following using receiver operating curve (ROC) analysis using visual read of [18F]-Flutemetamol PET scans as the standard of truth. We used cut-offs modified from Sjogren, Vanderstichele^40^ for P-tau (≥ 80 pg/ml) and T-tau (> 300 pg/ml < 50 years; > 450 pg/ml 50–69 years; > 500 pg/ml for ≥ 70 years).

### Study design

**Figure 1:**
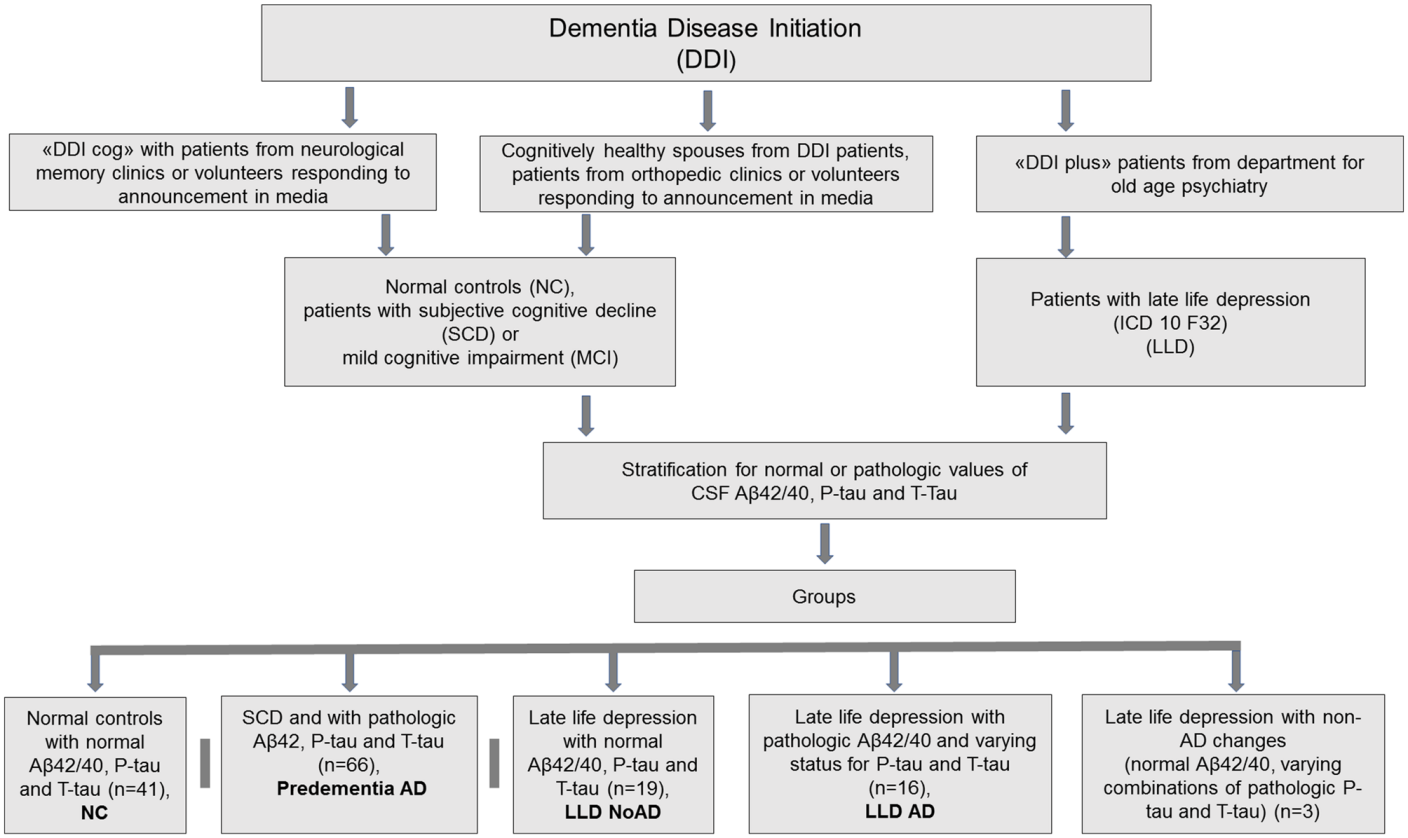
Inclusion of participants and groups.

A total of 145 participants were included for the present study, whereof 107 patients and controls were included from the DDI cog cohort, and 38 cases with LLD from the DDI plus cohort. Stratification by A/T/N yielded the following groups: NC group with normal cognition and normal AD biomarkers (n = 41, A-/T-/N-); a “Predementia AD” group comprising SCD and MCI patients with pathologic AD biomarkers (n = 66, A+/T+/N+); we stratified LLD patients into those with normal AD biomarkers (LLD NoAD; n = 19, A−/T−/N−,) those on the AD continuum (LLD AD; n = 16, A+/T*/N* where “*” indicates a positive or negative parameter) for our main analyses. We excluded LLD patients with non-AD pathologic changes (n = 3, A−/T*/N*) from further analyses, because the focus of this study was to explore possible effects of AD pathology on synaptic function and because of the group size. A flowchart (Fig. 1) and a table with detailed A/T/N characteristics for the LLD groups are provided (Table 1). The prevalence of LOD and anxiety in the LLD groups is provided (Table 2).

**Table 1:** Combinations of CSF AD parameters in the LLD group.

Normal AD biomarkers *“LLD NoAD”*	Alzheimer’s continuum *“LLD AD”*			Non-AD pathologic change		
A−/T−/N−	A+/T−/N−	A+/T−/N+	A+/T+/N+	A−/T+/N−	A−/T−/N+	A−/T+/N+
19 (50%)	7 (18.4%)	2 (5.3%)	7 (18.4%)	1 (2.6%)	1 (2.6%)	1 (2.6%)

A+ = pathologic Amyloid beta42/40; T+ = pathologic phosphorylated Tau; N+ = pathologic marker of neurodegeneration (here total Tau).

**Table 2:** Prevalence of Late-onset depression and comorbid anxiety in the LLD groups. Late-onset was defined when LLD occurred after the age of 60. Anxiety was defined in accordance to ICD10 (F41).

	LLD NoAD (n = 19)	LLD AD (n = 16)	Non-AD pathologic change (n = 3)
Late-onset (%within group)^1)^	52.6 (n = 10)	75.0 (n = 12)	66.7 (n = 2)
Comorbid anxiety (%within group)^2)^	36.8 (n = 7)	43.8 (n = 7)	0.0 (n = 0)

Chi square for 1) 1.9 (*p* = 0.40), for 2) 2.1 (*p* = 0.35).

### Statistics

Statistical analyses were performed with SPSS v 26 and R version 3.6.3. For between-group comparisons of demographics and clinical characteristics, one-way ANOVA was used for variables with assumed normal distributions (age, years of education and demographically adjusted T-scores from CERAD memory recall, TMT-B, COWAT and VOSP silhouettes) and the Kruskal–Wallis test was used for variables with non-normal distributions (MMSE and GDS). Differences in sex distributions were assessed with a chi-square test. For CSF measures, we fitted ANCOVA models with age and sex as covariates. In total, 6 ANCOVAS were carried out since we assessed 6 CSF markers (Aβ42/40, P-tau, T-tau, Ng, BACE1, Ng/BACE1). In preliminary models, treatment with antidepressants was included as a covariate but was omitted for reasons of parsimony since it had no significant effect. All except the Ng/BACE1 ratio and Aβ42/40 ratio showed distributions with kurtosis and/or skewness that led to violations of assumptions of ANCOVA analyses and were thus log-transformed prior to final analyses. If Kruskal–Wallis tests, ANOVA or ANCOVA models showed significant group differences (*p* < 0.05), planned comparisons between groups in accordance with our hypotheses were carried out: (1) symptom groups were compared to the NC group, (2) the LLD noAD group was compared to the LLD AD group. However, to reduce the family wise error rate associated with multiple testing of the same synapse markers individually (Ng and BACE1), and as a ratio (Ng/BACE1) the Holm-Bonferroni sequential procedure was used for these planned comparisons (twelve comparisons in total). The adjusted *p*-values (p_ADJ_) are in accordance with significance thresholds following this procedure.

## Results

The LLD patients emerged as a heterogenous group with respect to AD biomarkers based on A/T/N index: LLD NoAD (n = 19), LLD AD (n = 16) and LLD with non-AD typical changes (n = 3 which we did not include in further analyses due to our hypotheses and research questions) (Table 1). Details pertaining to between group comparisons of demographics, clinical and CSF markers are shown in Table 3. NC were significantly younger than all other groups (NC vs predementia AD *p* < 0.01; NC vs LLD NoAD *p* < 0.0001; NC vs LLD AD *p* < 0.001) and had significantly more education years than LLD NoAD (*p* < 0.0001). The LLD groups and predementia AD showed significantly higher GDS scores than NC (*p* < 0.0001). Both LLD groups showed significantly poorer performance than NC in memory (CERAD recall T-score; LLD NoAD *p* < 0.0001; LLD AD *p* < 0.001) and executive function (TMT-B: LLD NoAD *p* < 0.001; LLD AD *p* < 0.001); in addition, the LLD AD group showed poorer visuospatial performance than NC (VOSP *p* < 0.01). None of these parameters showed significant differences between the LLD groups. Late-onset LLD was differently prevalent (*p* = 0.40) in the LLD groups (LLD AD 75.0%; non-AD pathological changes 66.7%; LLD NoAD 52.6%). Anxiety as a comorbidity was more prevalent (*p* = 0.35) in the LLD AD group (43.8%) than in the LLD NoAD (36.8%) and non-AD pathological changes (0%) groups.

**Table 3:** Between-group comparisons of CSF biomarkers, demographics, clinical including neuropsychological data.

Variable	Groups				*F/χ*^*2*^*/H/η*^*2*^*/η*_*p*_^*2*^*/(P)*	Planned comparisons (P)			
	1. NC n = 41	2. Predementia AD n = 66	3. LLD NoAD n = 19	4. LLD AD n = 16		1 versus 2	1 versus 3	1 versus 4	3 versus 4
**Age**									
Mean (SD)	64 (4.8)	67.9 (8.0)	71.9 (6.9)	72.9 (6.8)	*F*(3,136) = 8.7 *η*^*2*^ = 0.16 **(< 0.0001)**	**< 0.01**	**< 0.0001**	**< 0.001**	*n.s*
**Male**									
n (%)	18 (43.9)	38 (57.6)	8 (42.1)	6 (37.5)	*χ*^*2*^ = 3.6 (n.s)	*	*	*	*
**Education years**									
Mean (SD)	14.4 (3.2)	13.2 (3.2)	11.3 (2.4)	12.7 (3.2)	*F*(3,136) = 4.7 *η*^*2*^ = 0.09 **(< 0.01)**	*n.s*	**< 0.0001**	*n.s*	*n.s*
**GDS**									
Median (IQR)	0 (1)	2.0 (3)	5.0 (6)	5.0 (4)	*H*(3) = 47.9, *η*^*2*^ = 0.33 **(< 0.0001)**	**< 0.0001**	**< 0.0001**	**< 0.0001**	*n.s*
**MMSE**									
Median (IQR)	29 (2)	27 (4)	29 (5)	29 (5)	*H*(3) = 22.6 *η*^*2*^ = 0.14 **(< 0.0001)**	**< 0.0001**	*n.s*	*n.s*	*n.s*
**CERAD Recall T**									
Mean (SD)	54.6 (8.0)	35.0 (13.1)	40.9 (11.5)^(n=18)^	42.9 (8.9)	*F*(3,137) = 26.1 *η*^*2*^ = 0.36 **(< 0.0001)**	**< 0.0001**	**< 0.0001**	**< 0.001**	*n.s*
**TMT-B T**									
Mean (SD)	48.6 (9.1)^(n=40)^	37.6 (14.1)	35.0 (18.1)^(n=16)^	34.6 (15.4)^(n=14)^	*F*(3,132) = 7.4 *η*^*2*^ = 0.15 **(< 0.0001)**	**< 0.0001**	**< 0.001**	**< 0.001**	*n.s*
**COWAT T**									
Mean (SD)	52.8 (9.5)^(n=40)^	48.9 (10.1)	44.9 (11.5)^(n=16)^	48.3 (7.3)^(n=15)^	*F*(3,133) = 2.8 (n.s)	*	*	*	*
**VOSP T**									
Mean (SD)	54.0 (10.2)^(n=40)^	48.2 (10.4)^(n=62)^	49.5 (9.4)^(n=18)^	45.7 (11.3)^(n=15)^	*F*(3,131) = 3.4 *η*^*2*^ = 0.07 **(< 0.05)**	**< 0.01**	*n.s*	**< 0.01**	*n.s*
**CSF Aβ42/40 ratio**									
Mean (SD)	0.101 (0.101)	0.047 (0.008)	0.096 (0.010)	0.051 (0.014)	*F*(3,136) = 325.4 *η*_*p*_^*2*^ = 0.88, **(< 0.0001)**	**< 0.0001**	**< 0.05**	**< 0.0001**	**< 0.0001**
**CSF P-tau**									
Mean (SD)	50.6 (13.1)	118.2 (34.5)	51.9 (11.7)	80.4 (32.2)	*F*(3,136) = 90.6, *η*_*p*_^*2*^ = 0.67, **(< 0.0001)**	**< 0.0001**	*n.s*	**< 0.0001**	**< 0.0001**
**CSF T-tau**									
Mean (SD)	289.0 (88.0)	858.3 (248.9)	300.5 (76.3)	576.9 (271.4)	*F*(3,136) = 121.2 *η*_*p*_^*2*^ = 0.73 **(< 0.0001)**	**< 0.0001**	*n.s*	**< 0.0001**	**< 0.0001**
**CSF Ng**									
Mean (SD)	303.8 (107.1)	559.8 (195.5)	264.4 (83.9)	508.9 (356.1)	*F*(3,136) = 35.2 *η*_*p*_^*2*^ = 0.44 **(< 0.0001)**	**< 0.0001**^**a**^	*n.s*^a^	**< 0.05**^**a**^	**< 0.001**^**a**^
**CSF BACE1**									
Mean (SD)	2205.7 (585.9)	2847.4 (770.2)	2211.2 (600.5)	2849.8 (1000.3)	*F*(3,136) = 8.9 *η*_*p*_^*2*^ = 0.16 **(< 0.0001)**	**< 0.01**^**a**^	*n.s*^a^	*n.s*^a^	**< 0.05**^**a**^
**CSF Ng/BACE1**									
Mean (SD)	0.137 (0.03)	0.197 (0.04)	0.123 (0.03)	0.167 (0.05)	*F*(3,136) = 34.4 *η*_*p*_^*2*^ = 0.43 **(< 0.0001)**	**< 0.0001**^**a**^	*n.s*^a^	**< 0.05**^**a**^	**< 0.01**^**a**^

*n* = *sample size; n.s.* = *non-significant results;* *no comparisons performed*; F* = *F-statistic; χ*^*2*^ = chi-square *H* = Kruskal–Wallis test; *η*^*2*^ = eta-squared; *η*_*p*_^*2*^ = partial eta squared; P = p-value; IQR = interquartile range.

^a^*p* values adjusted (p_ADJ_) for familywise multiple testing according to Holm–Bonferroni.

### CSF Ng and BACE1

**Figure 2:**
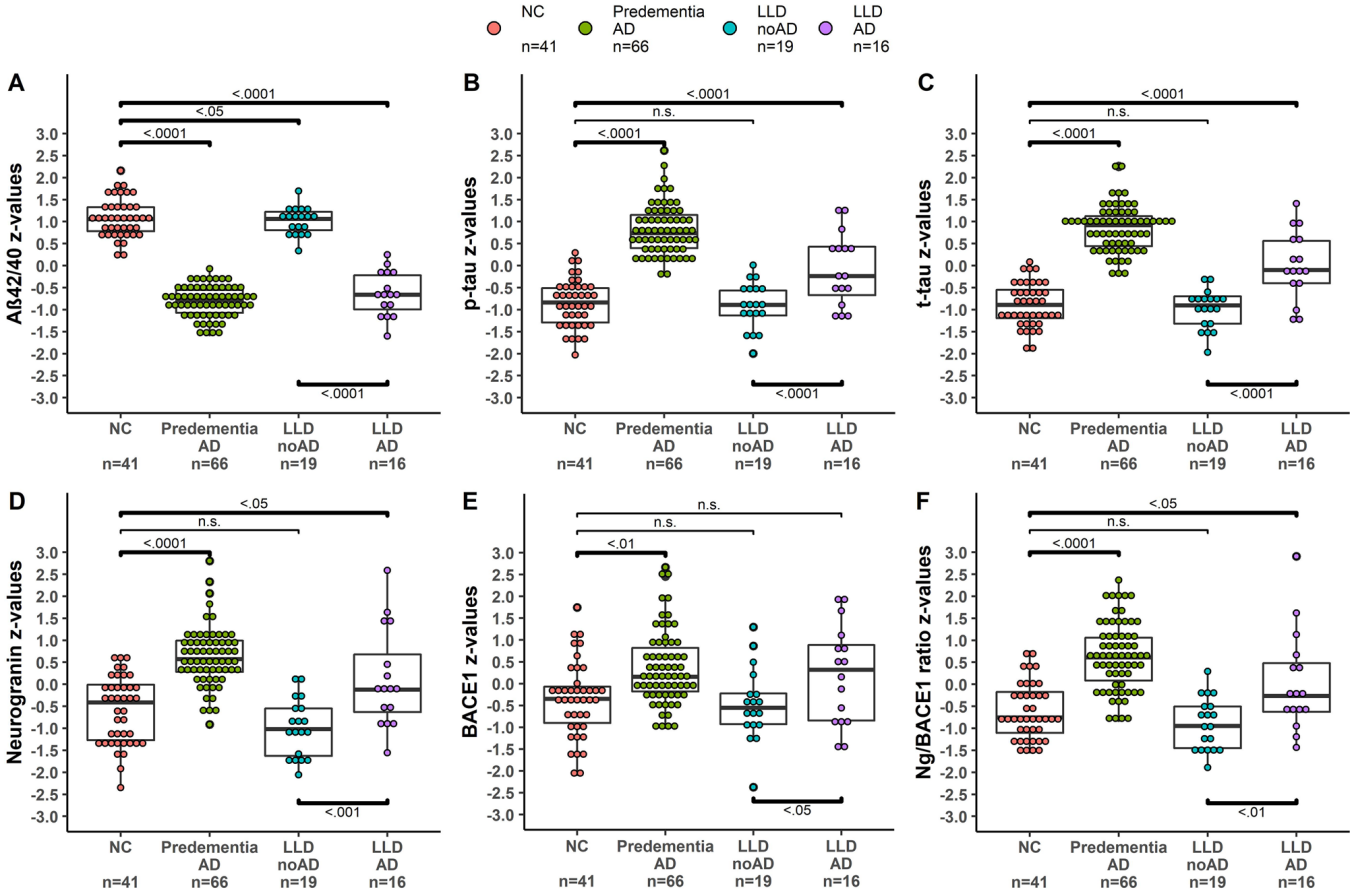
Scatter dot plots for CSF concentrations of (**A**) Aβ42/40 ratio, (**B**) P-tau and (**C**) T-tau (CSF markers for AD pathology) and of (**D**) neurogranin (Ng), (**E**) BACE1 and (**F**) Ng/BACE1 ratio (CSF Synapse biomarkers). The lines in the plots represent the mean values, the bars the standard deviation. Statistical significance at *p* < 0.05; n.s. = non significant. NC = normal controls; predementia AD = predementia Alzheimer’s disease; LLD NoAD = late life depression without AD pathology; LLD AD = late life depression with AD pathology (pathologic Aβ42/40 ratio, varying combinations of pathologic P-tau and T-tau).

Details pertaining to between-group analyses are shown in Table 3. LLD AD and predementia AD showed significantly higher Ng levels than NC (LLD AD, p_ADJ_ < 0.05; predementia AD, p_ADJ_ < 0.0001); LLD NoAD showed Ng levels similar to NC. BACE1 levels were altered in a parallel fashion: LLD AD and predementia AD showed higher BACE1 levels than NC (LLD AD, n.s.; predementia AD, p_ADJ_ < 0.01); LLD NoAD showed BACE1 levels similar to NC. The Ng/BACE1 ratio followed the same pattern (LLD AD vs NC p_ADJ_ < 0.05; predementia AD vs NC p_ADJ_ < 0.0001; LLD NoAD vs NC n.s.) (Fig. 2).

Notably, the depression groups LLD AD and LLD NoAD showed significant differences in Ng, BACE1 and Ng/BACE1 ratio with higher levels for Ng (p_ADJ_ < 0.001), BACE1 (p_ADJ_ < 0.05) and the Ng/BACE1 ratio (p_ADJ_ < 0.01) in the LLD AD group.

### CSF Aβ42/40 ratio, P-tau and T-Tau

Details pertaining to between-group analyses are shown in Table 3. All patient groups had significantly lower Aβ42/40 ratio than NC (predementia AD and LLD AD, *p* < 0.0001; LLD NoAD, *p* < 0.05). P-tau and T-tau were significantly higher in predementia AD and LLD AD than in NC (*p* < 0.0001), but similar in NC and LLD NoAD. The two LLD groups exhibited significant differences in all three parameters: the LLD AD group showed significantly lower (*p* < 0.0001) Aβ42/40 ratio and significantly higher (*p* < 0.0001) P-tau and T-tau levels than LLD NoAD.

## Discussion

The mechanisms of LLD are unclear. While LLD shares clinical features with predementia AD, it is unclear to which extent this reflects shared biological features. Here we could show that according to A/T/N index, some LLD patients (LLD AD) exhibited pathological AD markers thus sharing CSF findings with predementia AD patients and being on the AD biocontinuum^41^, while other LLD patients (LLD NoAD) showed normal AD biomarkers closer to NC. Interestingly, although in normal ranges, the CSF Aβ42/40 ratio was significantly lower in LLD NoAD than in NC. We could support our main hypothesis that CSF Ng and BACE1 levels would depend on AD status in LLD. LLD AD patients had significantly higher Ng and BACE1 levels than LLD NoAD patients and exhibited changes of these synaptic proteins parallel to those in predementia AD patients, albeit to a lesser extent. LLD NoAD patients, on the other hand, exhibited levels of Ng, BACE1 and Ng/BACE1 ratio similar to NC. The neuropsychological performance of both LLD groups was significantly poorer than that of NC, with poorer memory function (CERAD recall), poorer executive function (TMT-B) in both LLD groups and, in the LLD AD group, also poorer visuospatial performance (VOSP). A smaller portion of the LLD patients showed non-AD pathologic changes, exhibiting normal Aβ42/40 levels, but pathological P-tau and N for neurodegeneration or a combination of both (A-/T*/N*). In these patients, other mechanisms such as tauopathies or other neurodegenerative diseases may be associated to LLD. LLD may appear as a recurrent disease after debut in earlier life (EOD) or for the first time in later life (LOD)^25^. Biological differences between EOD and LOD have been discussed^25,42^, which is in line with our findings of a higher prevalence of LOD in the LLD AD group. We could further show that a larger portion in the LLD AD group than in the LLD NoAD group had comorbid anxiety, which concurs with the assumption that anxiety may be a prodrome or a risk-factor of AD^43,44^.

The association between Aβ pathology and LLD is unclear and results are conflicting^42^. In our sample, all LLD patients had significantly lower CSF Aβ42/40 ratios than NC, although the LLD NoAD group was within normal ranges. Since we could show that BACE1 levels were normal in LLD NoAD, other mechanisms than BACE1 activity likely account for the lowered Aβ42/40 ratio in LLD NoAD, putatively linked to depressive symptoms^45^. It has been speculated^46^ that lower Aβ in LLD follows a decreased neuronal activity^47^ rather than amyloid plaque formation as is the case in AD. Lower, but still normal CSF concentrations of Aβ42/40 in the LLD NoAD group may suggest incipient AD linked mechanisms or other factors leading to lower CSF Aβ42/40 in LLD NoAD. Overproduction of Aβ has been hypothesized^48^ to be a repair mechanism for neuronal dysfunction in the context of AD and depression. The lack of significant differences in P-tau and T-tau concentrations between LLD AD NoAD and NC indicates that tau-pathology or neurodegeneration are not features of LLD NoAD. Our results for levels of Ng and BACE1 in LLD are partly in line with other results^16,16^ of lower Ng levels in LLD than in AD. Neither of these studies, however, stratified LLD for possible AD pathology. Therefore, concluding from our finding of a LLD NoAD group, a possible explanation for their findings^16,16^ of lower Ng and BACE in LLD than in AD may be that a subset of their patients had normal AD biomarkers shifting the levels of Ng and BACE to statistically lower levels than in AD. While our findings of Ng and BACE in the LLD NoAD group provide no evidence for synaptic dysfunction in LLD, there is other evidence of synaptic dysfunction in LLD. For example, LLD may be associated with reduced synaptic density^49^. Chronic stress, as in LLD, may lead to reduced spine density in the limbic system^50^, a central region for emotional regulation. Possibly, synaptic dysfunction in the hippocampus contributes to both affective and cognitive symptoms in depression^49^, which would be compatible with poorer memory in our LLD groups.

Showing lower cognitive performance and signs of AD pathology without dementia, the LLD AD group can be conceptualized as “Predementia AD with depression”. It has been suggested^51^ to describe an “AD positive” subtype of LLD. However, we would suggest the other way around to describe a subtype “Predementia AD with depression” since it cannot be excluded that the AD pathology contributes to depression in this group in the sense of “neuro”-psychiatric symptoms. This concurs with recent work demonstrating that mild psychiatric symptoms may represent prodromal AD (“mild behavioural impairment”)^43^ and with the assumption^52^ that psychiatric phenotypes can be indistinguishable clinically while they differ etiologically. Our finding of lower Aβ levels in LLD AD and poorer visuospatial performance is in line with previous findings^42^. Poorer visuospatial performance may be associated to pathology in the parietal lobe in early AD^53^. A relatively new biomarker strongly associated to cognitive performance in AD but also to differentiate AD from other underlying pathologies is the Ng/BACE ratio^54^. It has been shown that higher values of the Ng/BACE are associated with poorer memory performance in predementia AD patients^55^. This is in line with a significantly higher Ng/BACE ratio and poorer memory performance in our LLD AD group. The LLD NoAD group also performed poorer in memory function and executive function (TMT-B) than NC, but showed no significant difference to NC in visuospatial abilities, which may be associated to normal AD biomarker status in the LLD NoAD group. Probably, in LLD NoAD other mechanisms than those leading to alterations in Ng or BACE1 drive the cognitive symptoms. As amyloid precursor protein function, and Aβ-metabolism is linked to synaptic plasticity and learning, the cognitive symptoms in the LLD NoAD group may be linked to Aβ metabolism reflected in the lower, albeit normal levels of Aβ 42/40 ratio. Poorer cognitive performance in LLD NoAD is generally in line with previous results and clinical criteria implying that cognitive symptoms are an integral part of depression^19,56^.

Limitations of this study include the cross-sectional design, which is why possible causal or temporal associations of clinical and CSF markers cannot be ascertained. Of note, data are being collected and the longitudinal course will be described in subsequent papers. It is still unclear how plasma levels of Aβ, P-tau and T-tau correlate with central-nervous levels of Aβ, P-tau and T-tau. A high correlation of plasma Aβ, P-tau and T-tau to respective PET findings has been reported^57^. We will address the relation of AD markers in plasma and CSF in a subsequent study. We will augment the included CSF markers with alpha synuclein and other synaptic markers. Another limitation is the rather small sample size, which is also why we did not further categorize the LLD AD group (LLD AD A+/T+/N+ etc.). The GDS scores in our LLD groups were low, which may be explained by inclusion after discharge and that patients with more severe depression were often too frail to be included or to give informed consent. Our LLD groups were significantly older than the NC. We see this as a limitation in a study into age-related disorders and included age as a covariate.

A strength of the study lies in the biologically defined groups based on CSF AD pathology, enabling us to study synaptic markers in a LLD NoAD group which is unbiased from AD pathology. The value of our findings lies in the demonstration of distinct LLD groups where one showed no AD pathology and synaptic markers similar to NC, while the other showed a typical AD profile including characteristic changes of synaptic markers. Moreover, we assessed Aβ alterations both continuously and dichotomized. Although pseudodepression and pseudodementia are being discussed controversially^56^, our findings may be of value in the discussion of these concepts.

In conclusion, we could demonstrate that LLD is heterogenous with respect to AD pathology. We could support our main hypothesis and found higher Ng, BACE1 and Ng/BACE1 ratio in LLD AD than in LLD NoAD and NC. This finding suggests that Ng and BACE1 in LLD depend on AD status as has been reported typical for AD. Similar Ng and BACE levels in LLD NoAD and NC indicate that these markers do not directly reflect mechanisms underlying depression in the LLD NoAD group. Our findings suggest associations of Aβ metabolism to LLD. Both LLD groups showed significantly lower Aβ42/40 ratios than NC. The LLD AD group showed pathological Aβ42/40 levels and AD typical Ng and BACE1 alterations, which is why he LLD AD group may be conceptualized as a “predementia AD with depression” group. The LLD NoAD group showed Aβ42/40 levels lower than in NC but in normal ranges which may suggest that mechanisms other than in AD are involved. Examination of CSF AD pathology in LLD patients should be considered on a routine basis since predementia AD may be associated. It has been reported that Aβ levels may predict poorer response to antidepressant treatment^51^. Except in visuospatial performance, the LLD groups are clinically similar, while they differ in CSF findings. This may indicate that different mechanisms drive the clinical signs in our LLD groups. Our study may contribute to a better understanding of LLD underlying mechanisms and to a better conceptualization of LLD considering clinical overlap with predementia AD. The clinical implications of our findings lie in the CSF based identification of LLD patients with a higher risk of developing AD dementia.

## Supplementary Information

Supplementary Information.
