# Author Correction: Cerebrospinal fluid markers for synaptic function and Alzheimer type changes in late life depression

**DOI:** 10.1038/s41598-022-22274-1

**Published:** 2022-10-18

**Authors:** Nikias Siafarikas, Bjørn-Eivind Kirsebom, Deepak P. Srivastava, Cecilia M. Eriksson, Eirik Auning, Erik Hessen, Geir Selbaek, Kaj Blennow, Dag Aarsland, Tormod Fladby

**Affiliations:** 1 Department of Geriatric Psychiatry, Akershus University Hospital, Sykehusveien 25, 1478 Lørenskog, Norway; 2 Faculty of Medicine, University of Oslo, Oslo, Norway; 3 Department of Neurology, University Hospital of North Norway, Tromsø, Norway; 4 Department of Psychology, Faculty of Health Sciences, UiT The Arctic University of Norway, Tromsø, Norway; 5 Department of Basic and Clinical Neuroscience, Institute of Psychiatry, Psychology and Neuroscience, King’s College London, London, SE5 9NU UK; 6 Department of Neurology, Akershus University Hospital, Lørenskog, Norway; 7 Department of Psychology, University of Oslo, Oslo, Norway; 8 Norwegian National Advisory Unit On Aging and Health, Vestfold Hospital Trust, Tønsberg, Norway; 9 Department of Geriatric Medicine, Oslo University Hospital, Oslo, Norway; 10 Department of Psychiatry and Neurochemistry, Institute of Neuroscience and Physiology, The Sahlgrenska Academy, University of Gothenburg, Gothenburg, Sweden; 11 Clinical Neurochemistry Laboratory, Sahlgrenska University Hospital, Mölndal, Sweden; 12 Department of Old Age Psychiatry, Institute of Psychiatry, Psychology and Neuroscience, King’s College London, London, SE5 8AF UK; 13 Institute of Clinical Medicine, University of Oslo, Campus Ahus, Oslo, Norway

Correction to: *Scientific Reports* 10.1038/s41598-021-99794-9, published online 13 October 2021

The original version of this Article omitted an affiliation for Nikias Siafarikas. The correct affiliations are listed below.

Department of Geriatric Psychiatry, Akershus University Hospital, Sykehusveien 25, 1478 Lørenskog, Norway.

Faculty of Medicine, University of Oslo, Oslo, Norway.

The original Article has been corrected.

