# Supplementary material for: Cerebrospinal fluid markers for synaptic function and Alzheimer type changes in late life depression
Source: Sci Rep. 2021 Oct 13;11:20375. doi: 10.1038/s41598-021-99794-9 (PMC8514484; doi:10.1038/s41598-021-99794-9)
Supplement: Supplementary file 1 — Supplementary Information. [file 41598_2021_99794_MOESM1_ESM.docx]

**Supplementary material

Mesoscale Aβ_42_ and Aβ_40/42_ cut-offs according to 18F-flutemetamol PET visual read**

The concentration ratio of cerebrospinal fluid (CSF) Aβ_42_ to Aβ_40_ has been shown to improve diagnostic accuracy for amyloid plaque pathology as compared to using Aβ_42_ alone^1^. To compare the diagnostic accuracies and determine optimum cut-off values for the mesoscale discovery (MSD) CSF Aβ_42_ and Aβ_42/40_ ratio assays, n=83 cases and controls from the dementia disease initiation (DDI) cohort with ^18^F-Flutemetamol amyloid PET scans were included. The sample comprised n=9 (12.7%) healthy controls, n=44 (62%) Subjective Cognitive Decline (SCD), n=15 (22.5%) Mild Cognitive Impairment (MCI) and n=2 (2,8%) cases with dementia.
Using receiver operating curve (ROC) analysis, we determined the AUC, sensitivity and specificity and optimum cut-off values for the respective CSF biomarkers based on visual read of the PET images by trained radiologists as the standard of truth. Lastly, the resulting ROC curves were compared with a one-tailed Delong’s test for two correlated ROC curves ^2^ hypothesizing that Aβ_42_ AUC would be smaller than the Aβ_42/40_ ratio AUC.

**Results and conclusions**Detailed results are summarized in supplementary table 1 and supplementary figure 1.
The diagnostic accuracy of CSF MSD Aβ_42/40_ ratio (AUC: .957) was superior to CSF MSD Aβ_42_ (AUC: .867) against 18F-flutemetamol PET visual read as the standard of truth (*z*=-2.5, *p*<.01). ROC results were in accordance with previous reports using the MSD Aβ_42_ and Aβ_40_ assays against Amyloid PET ^3,4^.

| **Supplementary table 1.** ROC analyses of MSD CSF Aβ_42/40_ against ^18^F-Flutemetamol PET | | | | | | | |
| --- | --- | --- | --- | --- | --- | --- | --- |
| **Standard of truth** | **CSF Measure** | **AUC**  **(95 % CI)** | **Aβ+/ Aβ-** | **Spec.** | **Sens.** | **Cut-off** | **Delong’s test (z/p)** |
| Visual read | MSD  CSF Aβ_42/40_ ratio | .957  (.903-1) | 29/54 | .963 | .931 | ≤.077 | -2.5 **(<.01)** |
| Visual read | MSD  CSF Aβ_42_ | .867  (.793-.944) | 29/54 | .778 | .828 | ≤.521 |  |
| *Notes.* MSD= Mesoscale Discovery; CSF= Cerebrospinal fluid; AUC = Area Under curve; CI= confidence interval. | | | | | | | |

**
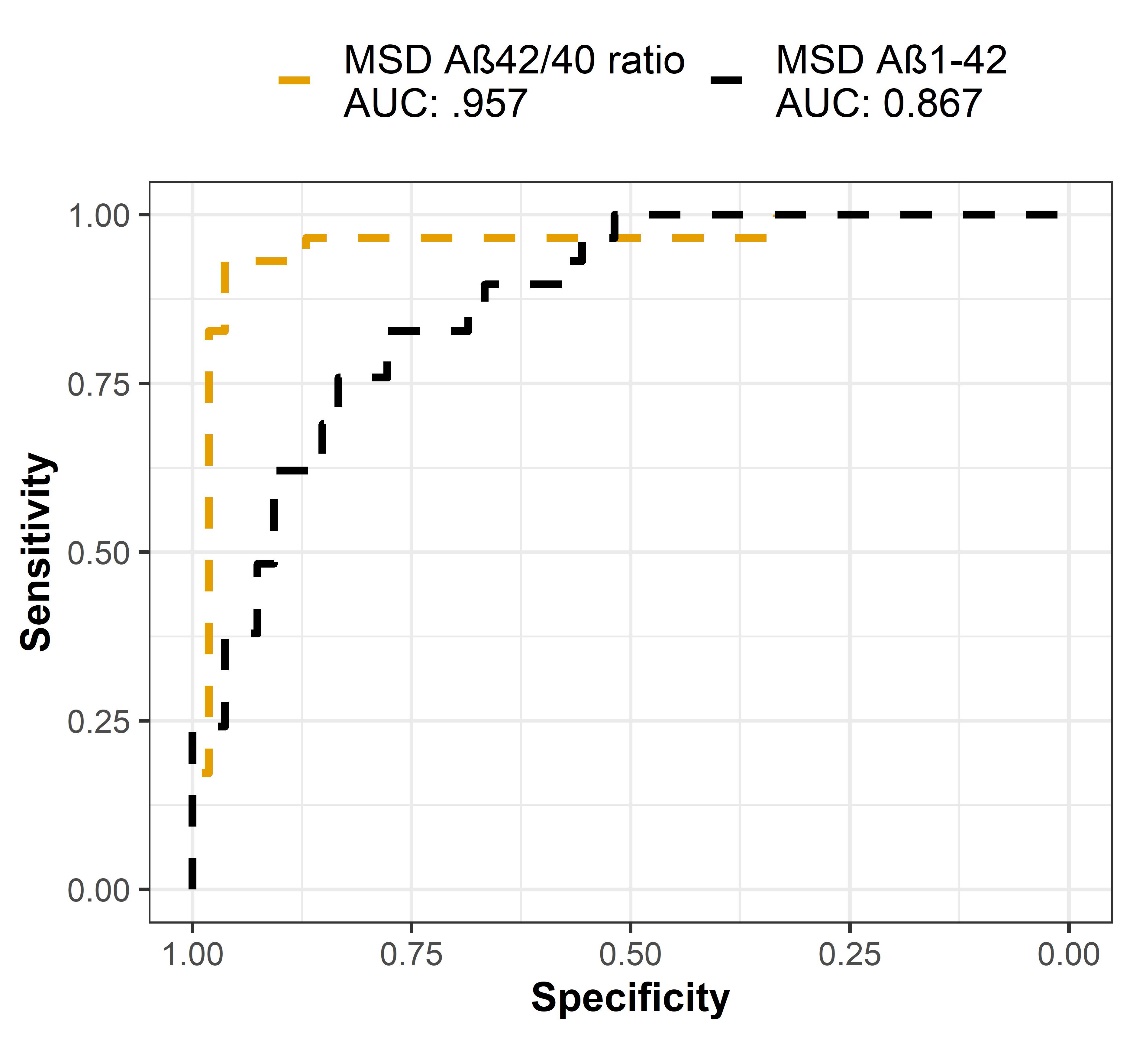
**

**Supplementary figure 1.** ROC curve comparisons of MSD CSF Aβ_42_ and Aβ_42/40_ ratio against visual read of ^18^F-flutemetamol PET

1 Hansson, O., Lehmann, S., Otto, M., Zetterberg, H. & Lewczuk, P. Advantages and disadvantages of the use of the CSF Amyloid β (Aβ) 42/40 ratio in the diagnosis of Alzheimer's Disease. *Alzheimer's research & therapy* **11**, 34, doi:10.1186/s13195-019-0485-0 (2019).

2 Robin, X. *et al.* pROC: an open-source package for R and S+ to analyze and compare ROC curves. *BMC bioinformatics* **12**, 77, doi:10.1186/1471-2105-12-77 (2011).

3 Janelidze, S. *et al.* CSF Aβ42/Aβ40 and Aβ42/Aβ38 ratios: better diagnostic markers of Alzheimer disease. *Ann Clin Transl Neurol* **3**, 154-165, doi:10.1002/acn3.274 (2016).

4 Janelidze, S. *et al.* Concordance Between Different Amyloid Immunoassays and Visual Amyloid Positron Emission Tomographic Assessment. *JAMA Neurol* **74**, 1492-1501, doi:10.1001/jamaneurol.2017.2814 (2017).
